# Measuring Local-Area Racial Segregation for Medicare Hospital Admissions

**DOI:** 10.1001/jamanetworkopen.2024.7473

**Published:** 2024-04-19

**Authors:** Ellesse-Roselee L. Akré, Deanna Chyn, Heather A. Carlos, Amber E. Barnato, Jonathan Skinner

**Affiliations:** 1 Department of Health Policy and Management, Bloomberg School of Public Health, Johns Hopkins University, Baltimore, Maryland; 2 The Dartmouth Institute for Health Policy, and Clinical Practice, Geisel School of Medicine at Dartmouth, Lebanon, New Hampshire; 3 Department of Economics, Dartmouth College, Hanover, New Hampshire; 4 Dartmouth Cancer Center, Lebanon, New Hampshire

## Abstract

**Importance:**

Considerable racial segregation exists in US hospitals that cannot be explained by where patients live. Approaches to measuring such segregation are limited.

**Objective:**

To measure how and where sorting of older Black patients to different hospitals occurs within the same health care market.

**Design, Setting, and Participants:**

This retrospective cross-sectional study used 2019 Medicare claims data linked to geographic data. Hospital zip code markets were based on driving time. The local hospital segregation (LHS) index was defined as the difference between the racial composition of a hospital’s admissions and the racial composition of the hospital’s market. Assessed admissions were among US Medicare fee-for-service enrollees aged 65 or older living in the 48 contiguous states with at least 1 hospitalization in 2019 at a hospital with at least 200 hospitalizations. Data were analyzed from November 2022 to January 2024.

**Exposure:**

Degree of residential segregation, ownership status, region, teaching hospital designation, and disproportionate share hospital status.

**Main Outcomes and Measures:**

The LHS index by hospital and a regional LHS index by hospital referral region.

**Results:**

In the sample of 1991 acute care hospitals, 4 870 252 patients (mean [SD] age, 77.7 [8.3] years; 2 822 006 [56.0%] female) were treated, including 11 435 American Indian or Alaska Native patients (0.2%), 129 376 Asian patients (2.6%), 597 564 Black patients (11.9%), 395 397 Hispanic patients (7.8), and 3 818 371 White patients (75.8%). In the sample, half of hospitalizations among Black patients occurred at 235 hospitals (11.8% of all hospitals); 878 hospitals (34.4%) exhibited a negative LHS score (ie, admitted fewer Black patients relative to their market area) while 1113 hospitals (45.0%) exhibited a positive LHS (ie, admitted more Black patients relative to their market area); of all hospitals, 79.4% exhibited racial admission patterns significantly different from their market. Hospital-level LHS was positively associated with government hospital status (coefficient, 0.24; 95% CI, 0.10 to 0.38), while New York, New York; Chicago, Illinois; and Detroit, Michigan, hospital referral regions exhibited the highest regional LHS measures, with hospital referral region LHS scores of 0.12, 0.16, and 0.21, respectively.

**Conclusions and Relevance:**

In this cross-sectional study, a novel measure of LHS was developed to quantify the extent to which hospitals were admitting a representative proportion of Black patients relative to their market areas. A better understanding of hospital choice within neighborhoods would help to reduce racial inequities in health outcomes.

## Key Points

**Question:**

Do local acute care hospitals admit representative proportions of Black Medicare fee-for-service beneficiaries based on their market areas?

**Findings:**

In the cross-sectional study using 1991 hospitals admitting 4 870 252 Medicare fee-for-service patients, 79.4% of the acute care hospitals in the sample did not admit a representative proportion of Black Medicare enrollees based on their market area.

**Meaning:**

These findings suggest that a strong degree of hospital segregation is still occurring in US hospitals even when controlling for residential segregation.

## Teaser

This cross-sectional study assesses local hospital segregation in US hospitals, defined as the difference between the racial composition of a hospital’s admissions and the racial composition of the hospital’s market, using Medicare data for admissions among older Black patients.

## Introduction

Racial inequities in health outcomes can be, in part, attributed to disparities in treatment and utilization arising from geographic variation in clinical practices,^1,2,3^ historical and systemic racism, implicit bias, social determinants of health, and poor access to care.^4,5,6,7^ There has been increasing attention to the role of lower-quality care in segregated communities leading to poor health outcomes.^8,9^ In the case of Black patients, it is less well understood whether higher rates of patient admissions occur because hospitals’ markets comprise segregated communities, or because patients from the same communities are admitted to different hospitals. Local patient sorting could be positive if patients disproportionately seek care at hospitals providing more affirming care, such as that captured in an index of hospital inclusivity.^10^ Differential patterns of admissions could also lead to poorer overall health care quality if patients forgo higher-quality hospitals primarily serving White patients because of historical segregation or negative perception.^8^ And while several efforts have been made recently to capture inclusivity,^10,11^ to our knowledge, there is no measure of hospital sorting within hospital-level markets that is both distinct from traditional measures of segregation and predetermined, hence insulated from hospital efforts to attract or avoid certain patients.

The objective of this study was to create a novel local hospital segregation (LHS) index that measures whether a hospital’s racial pattern of admission is significantly different from the racial composition of its market (as measured by driving time). We then estimate the association between the LHS index (outcome) and a variety of hospital-level factors, such as teaching, for-profit, or disproportionate-share hospital (DSH) status (exposures). While we focused on people racialized as Black for the development and demonstration of the LHS index functionality, the approach can be easily applied to diverse racial or ethnic groups of interest.

## Methods

This cross-sectional study was approved by the Dartmouth Committee for the Protection of Human Subjects, which waived the requirement for obtaining informed consent because the data were deidentified and not collected as part of the study. Use of the Medicare claims data was approved by Center for Medicare & Medicaid Services (CMS) under a data use agreement. This study followed the Strengthening the Reporting of Observational Studies in Epidemiology (STROBE) reporting guideline.

### Medicare Hospitalization Data

We used the 2019 Medicare Master Beneficiary Summary File, 2019 Medicare Provider Analysis and Review (MedPAR) file of inpatient data, and the 2019 American Hospital Association Survey to construct an LHS index. Our measure of LHS accepts the spatial patterns of where Medicare enrollees live, assuming the existence of pre-existing patterns of residential segregation. For each hospital in our sample, we assessed the composition of Medicare enrollees who live in the hospital’s market area (residential segregation) and then compared the racial composition of hospitalizations with the composition of the market area.

### Hospital Market Area

A hospital’s market area was defined as all zip codes with a centroid within a 30-minute driving distance from the hospital’s address.^12^ We acknowledge that patients must often rely on public transportation, making it difficult to seek care at distant hospitals. We also recognize that driving times do not capture the impact of environmental racism in creating structural barriers to accessing necessary resources in areas that are inhabited by a majority Black population and other people of color, including American Indian or Alaska Native, Asian, and Latine populations.^13^ For example, the presence of large expressways divides cities and creates challenges to being admitted to hospitals that are physically nearby but difficult to access, especially for people who do not have cars. A sensitivity analysis was conducted considering more local markets defined by a 15-minute driving distance in addition to considering a 1-hour driving distance to better capture driving distances in rural areas (eAppendix in Supplement 1). Drive times from each hospital’s geocoded street address to the zip code centroids were constructed using ArcGIS Pro 3.1 and ArcGIS StreetMap Premium North America 2020 Release 4 (Esri).

### Local Hospital Segregation

The segregation index comprises the difference between 2 measures. The first is the hospital ratio (HR_i_), or the fraction of the patient group of interest (in this case, Black patients) admitted to hospital *i*. To focus on local markets, the sample is limited to people living in the *z*(*i*) zip codes within the hospital’s market area:
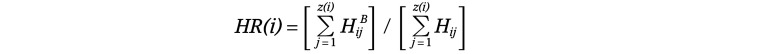
where *H_ij_^B^* is hospitalizations for Black enrollees in the *i*^th^ hospital in the *j*^th^ zip code and *H_ij_* is total hospitalizations. The market-level or residential fraction of the patient group of interest (Black) hospitalizations to total hospitalizations (ie, the market ratio [MR]) is written in a similar way:
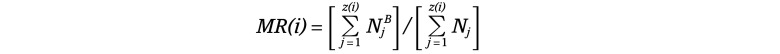
where N_j_^B^ is the number of hospitalizations for Black Medicare enrollees and *N_j_* is the total number of hospitalizations among Medicare enrollees living in zip code *j*, regardless of which hospital treated them.

The LHS index is the difference: *LHS(i)* = *HR(i)* − *MR(i)*. Values toward 1 correspond to a hospital serving a patient population that is primarily Black in a market area with few Black patients living there. Conversely, a value that tends toward −1 corresponds to a hospital in a market region comprising mostly Black enrollees whose patient population is primarily enrollees from other racial and ethnic groups. Within a market, the mean LHS index across hospitals will be approximately zero; this is because for every hospital with a higher-than-the-mean share of Black patients in the market, there must be another hospital with a lower-than-the-mean share (eAppendix in Supplement 1).

We note that this measure is flexible to a variety of specifications depending on the questions being asked. For example, a local segregation index can also be calculated for Asian or Hispanic hospital patients by simply changing the numerators in *HR(i)* and *MR(i)* to reflect the different patient populations; similarly, one may restrict the market (the denominator) to subgroups, for example Black and White patients only.

### Regional Measure of Local Segregation

While the LHS index is specific to each hospital, we also created a regional LHS measure to characterize the extent of local patient sorting in larger health care markets. This measure is the mean of the absolute value of the LHS index across the region, weighted by the size of the hospital. Following the Dissimilarity Index,^14^ this measure is the percentage of all hospitalizations in a region who must be moved across hospitals to achieve evenness or representativeness, but where the total number of hospitalizations at each hospital is held constant^8^ (eAppendix in Supplement 1). We adopt the Dartmouth Atlas hospital referral region (HRR) as our measure of regional markets.

### Study Sample

The study sample included Hispanic, non-Hispanic American Indian or Alaskan Native, non-Hispanic Asian or Pacific Islander, non-Hispanic Black (hereafter, *Black*), non-Hispanic White (hereafter, *White*), other (ie, participants who self-reported as other race or ethnicity with no further information), and unknown enrollees using the Research Triangle Institute race algorithm definitions.^15^ In the event of missing data, the Research Triangle Institute algorithm assumes racial and ethnic identity based on name, geography, and whether the person requested Social Security Administration and Medicare materials in Spanish. In this study, the Black racial category represents the group of individuals who were the direct target of historical segregation laws in the United States. We acknowledge that distinct analyses should be conducted for each racial and ethnic group to comprehensively understand the specific impacts of structural racism.

The sample comprises fee-for-service (FFS) Medicare enrollees aged 65 years and older who had a hospitalization in 2019. Hospitals included in the analysis met the following inclusion criteria: acute care or critical access hospital (does not include emergency hospitals) and located in the continental United States. We identified 4653 acute care or critical access hospitals (ie, nonemergency hospitals) providing treatment to any Medicare FFS beneficiaries in 2019 in the 48 contiguous states; 221 hospitals lacked the necessary geospatial data to construct the market area, leaving us with 4432 hospitals. We further limited the sample to hospitals with at least 200 total Medicare patients aged 65 years or older for statistical precision. Because of CMS suppression rules, we limited the sample to hospitals and market areas with at least 11 eligible Black patients and at least 11 eligible people not racialized as Black living in its market area. After using the utilization restrictions, we identified our sample of 1991 hospitals that met the inclusion criteria and were included in the analysis. The hospitals in the sample represent 4 870 252 patients and 7 349 194 hospitalizations (eAppendix in Supplement 1).

### Hospital Characteristics

Covariates included the hospital’s Census region, area racial composition (percentage of hospitalizations among Black patients in the market area), market share (the number of hospitals that are in a hospital’s market area), ownership (for profit, nonprofit, physician, or government), teaching hospital designation (teaching hospital or nonteaching hospital), and receipt of payment as a DSH (DSH hospital or non-DSH hospital). Prior literature has demonstrated that ownership, teaching hospital designation, and receipt of DSH payments are associated hospitals treating more Black patients.^16,17^ Geographic region and market share, or the density of hospitals in an area, have implications on the density of the Black population in a region. Seven hospitals with missing data on any of the hospital characteristics were dropped from the regression analysis but included in the overall analysis.

### Statistical Analysis

To judge whether a hospital’s racial composition of hospitalizations is significantly different from the market from which it draws patients, a fixed number of hospitalizations in hospital *i* (N_i_) was assumed. Under the null hypothesis of no local segregation, the racial mix of patients at the hospital should correspond to a random or representative sample of the market. The hospital’s racial composition is therefore distributed as a hypergeometric distribution. A 2-tailed Fisher exact test at α = .05 was used (with a χ^2^ approximation when N_i_ > 100 000). Univariate regressions to test the associations between hospital characteristics and the LHS outcome variable were conducted. All analyses were conducted using Stata version 17 (StataCorp) and SAS version 9.4 (SAS Institute) software. The data analysis was conducted between November 2022 and January 2024.

## Results

Among 1991 hospitals assessed, the mean (SD) LHS index was 0.02 (0.10) (range, −0.28 to 0.74). A total of 4 870 252 patients (mean [SD] age, 77.7 [8.3] years; 2 822 006 [56.0%] female) were treated, including 11 435 American Indian or Alaska Native patients (0.2%), 129 376 Asian patients (2.6%), 597 564 Black patients (11.9%), 395 397 Hispanic patients (7.8), and 3 818 371 non-Hispanic White patients (75.8%). There were 878 hospitals (44%) of hospitals with a negative LHS index (of which 684 were significantly negative, or 34.4% of all hospitals), meaning that they had proportionally fewer hospitalizations of Black patients relative to the Black residents living in surrounding area; 1117 hospitals (56%) had a positive LHS index (of which 896 were significantly positive, or 45.0% of all hospitals) (Table 1).

**Table 1.** Characteristics of Hospitals Treating Ever-Hospitalized Medicare Fee-for-Service Enrollees Aged at Least 65 Years, 2019

Characteristic	No. (%)		
	All hospitals	Positive LHS Index	Negative LHS Index
**Patient level^a^**			
Total, No.	5 040 259	2 747 587	2 292 672
Sex			
Female	2 822 006 (56.0)	1 532 382 (55.8)	1 289 624 (56.3)
Male	2 218 253 (44.0)	1 215 205 (44.2)	1 003 048 (43.8)
Race and ethnicity			
American Indian or Alaska Native	11 435 (0.2)	6785 (0.3)	4650 (0.2)
Asian or Pacific Islander	129 376 (2.6)	66 458 (2.4)	62 918 (2.7)
Black	597 564 (11.9)	445 709 (16.2)	151 855 (6.6)
Hispanic	395 397 (7.8)	221 431 (8.1)	173 966 (7.6)
Non-Hispanic White	3 818 371 (75.8)	1 961 334 (71.4)	1 857 037 (81.0)
Other^b^	37 501 (0.7)	19 723 (0.7)	17 778 (0.8)
Unknown	50 615 (1.0)	26 147 (1.0)	24 468 (1.1)
Age on January 1, 2019, mean (SD), y	77.66 (8.29)	77.33 (8.24)	78.07 (8.33)
**Hospital level **			
Total hospitals, No.	1991	1113	878
Disproportionate share hospital (DSH)	1755 (88.15)	1046 (93.98)	709 (80.75)
Census region			
Northeast	343 (17.2)	181 (16.3)	162 (18.5)
Midwest	415 (20.8)	226 (20.3)	189 (21.5)
South	849 (42.6)	483 (43.4)	366 (41.7)
West	384 (19.3)	223 (20.0)	161 (18.3)
Hospitals in market, No.^c^			
1-2	307 (15.4)	225 (20.2)	82 (9.3)
3-6	663 (33.3)	387 (34.8)	276 (31.4)
≥7	1021 (51.3)	501 (45.0)	520 (59.2)
Hospital ownership			
Private or religious (nonprofit)	1157 (58.1)	644 (57.9)	513 (58.4)
Physician or other	203 (10.2)	105 (9.4)	98 (11.2)
Private (for profit)	383 (19.2)	197 (17.7)	186 (21.2)
Government	248 (12.5)	167 (15)	81 (9.2)
Teaching hospital^d^	1072 (53.8)	670 (60.2)	402 (45.8)
LHS index, mean (%)	1.77 (10.26)	6.81 (10.54)	−4.62 (4.97)

Abbreviation: LHS, local hospital segregation.

^a^ Beneficiaries may be counted more than once if they received care at multiple hospitals in the sample.

^b^ Includes individuals who self-reported other race and ethnicity with no further information provided.

^c^ A hospital’s market area is defined as zip codes whose centroids are within 30-minute driving distance from the hospital’s geocoded address and includes the profiled hospital (ie, no market is smaller than 1).

^d^ Seven hospitals had unknown status.

Among sample hospitals, 1348 (79.4%) rejected the null hypothesis that they were drawing patients randomly from their market; for the remaining 643 hospitals (20.6%) we were not able to reject the null of no difference. Table 2 provides the LHS index for 10 hospitals with either very high or very low measures; total Medicare FFS admissions (drawn from the 30-minute market) are also presented. The top 5 hospitals with the highest values of the 30-day LHS index had LHS indices ranging from 0.741 for East Orange General Hospital (New Jersey) to 0.6214 for the West Suburban Medical Center in Oak Park (Illinois); all results were significantly different from zero. Thus, the fraction of Black admissions in the East Orange General Hospital (0.883) exceeds the fraction of Black admissions in the hospital’s 30-minute market (0.142) by 0.741. For positive values of the LHS index, 15-minute LHS measures were attenuated (meaning that hospitals were more reflective of their local markets); but both 15-minute and 1-hour LHS measures were significantly associated with the 30-minute LHS index (Table 2).

**Table 2.** Highest and Lowest Hospital-Level LHS Scores, 2019

Hospital	City	Medicare admissions, No.	LHS^a^		
			30-min	15-min	60-min
Highest LHS					
East Orange General Hospital	East Orange, New Jersey	1512	0.741	0.535	0.743
Sinai-Grace Hospital	Detroit, Michigan	5108	0.665	0.284	0.771
Harlem Hospital Center	New York, New York	1597	0.656	0.532	0.634
Newark Beth Israel Medical Center	Newark, New Jersey	3663	0.644	0.386	0.647
West Suburban Medical Center	Oak Park, Illinois	1562	0.624	0.423	0.616
Lowest LHS					
Alton Memorial Hospital	Alton, Illinois	3008	−0.282	−0.006	−0.090
Piedmont Henry Hospital	Stockbridge, Georgia	4654	−0.271	−0.030	0.015
Henry Ford Wyandotte Hospital	Wyandotte, Michigan	5525	−0.268	−0.014	−0.114
Anderson Hospital	Maryville, Illinois	2713	−0.268	0.001	−0.098
Community Hospital	Munster, Indiana	8166	−0.261	−0.141	−0.095

Abbreviation: LHS, local hospital segregation.

^a^ The LHS is defined as the difference between the share of admissions at that hospital for Black patients minus the share of admissions for Black patients in the hospital’s market.

For hospitals with the lowest values for the LHS index, meaning that the fraction of Black admissions was lower than the market share, Alton Memorial Hospital (Illinois) had an LHS index of −0.282, and Henry Ford Wyandotte Hospital (Michigan), had an LHS of −0.268, using markets defined by a 30-minute driving distance; however, when LHS was measured using a 15-minute driving times, both hospitals had lower LHS indices (Alton Memorial Hospital, −0.006; Henry Ford Wyandotte Hospital, −0.014). This suggests that, for many markets with sharply demarcated neighborhood racial composition, the results were sensitive to how the market was defined. We also observed some hospitals (eg, Franciscan Health Hammond in Hammond, Indiana, and Ochsner Medical Center-Kenner in Kenner, Louisiana) where the 15-minute LHS was larger than the 30-minute LHS (Table 2).

**Figure 1.**
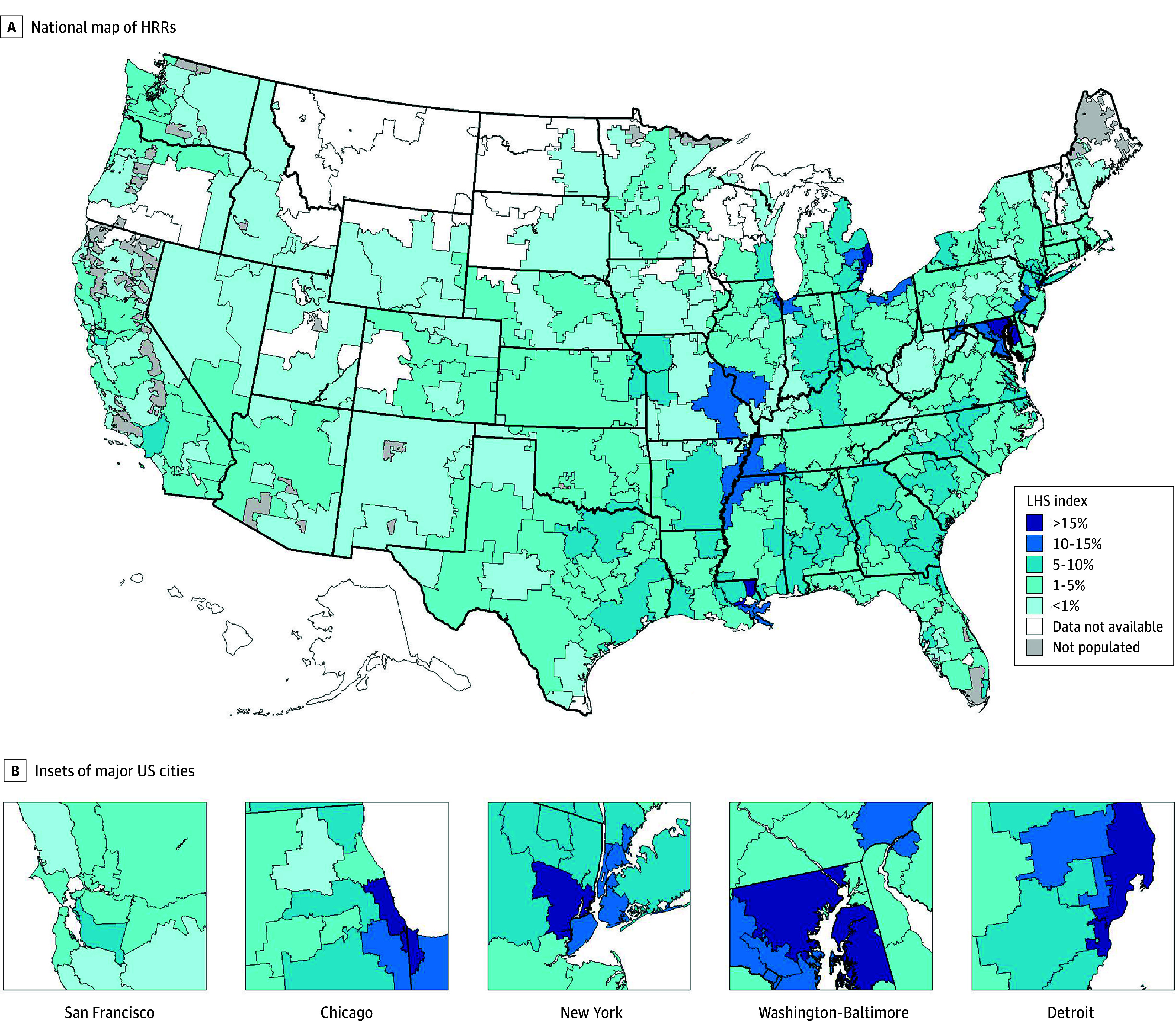
Regional Local Hospital Segregation (LHS) Index by Hospital Referral Region (HRR) in 2019 This regional measure takes the mean of the absolute values of the hospital-specific LHS, weighted by the hospital size; for 27 of the HRRs there were insufficient data to calculate the regional LHS.

There was considerable variation in the regional LHS measure, which is shown in Figure 1. In Detroit, Michigan, the regional LHS was 21.2%, meaning that 21.2% of patients would need to be moved within their market to attain representativeness of hospitals to their markets. Other areas with high rates of regional LHS were Newark, New Jersey (20.0%) and Chicago, Illinois (16.4%).

**Figure 2.**
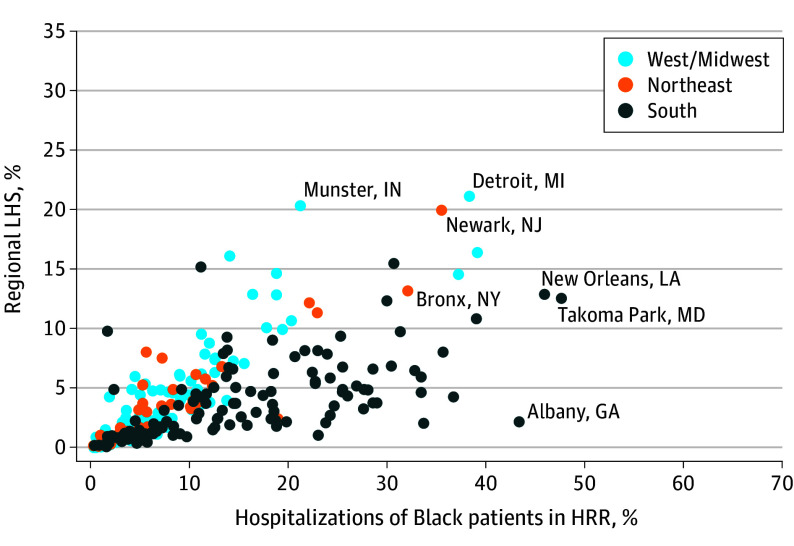
Regional Local Hospital Segregation (LHS) Index by Percentage of Hospitalizations for Black Patients in Health Referral Regions (HRRs) in 2019 Hospitalizations are among Black patients in the Medicare fee-for-service population in 2019. Each dot represents a hospital within an HRR.

While 31.2% of regions had a regional LHS of at least 5%, most regions exhibited very low rates of regional LHS, in most cases because of a low fraction of Black patients admitted to hospitals in these regions. Figure 2 demonstrates a positive association between the percentage of hospital admissions in the HRR for Black patients and the regional LHS. For example, there was much less within-region sorting of Black patients in Albany, Georgia, compared with Newark, New Jersey, despite Albany having a larger overall fraction of Black hospitalizations.

### Association of the LHS With Hospital-Specific Factors

Hospital-level LHS was associated with percentage of Black hospitalizations (in 10–percentage point increments) in the market area (coefficient, 0.15; 95% CI, 0.11 to 0.19), sharing a market with 7 or more hospitals (coefficient, 0.18; 95% CI, 0.05 to 0.31), government ownership (coefficient, 0.24; 95% CI, 0.10 to 0.38), being a teaching hospital (coefficient, 0.36; 95% CI, 0.28 to 0.45), and receiving DSH payments (coefficient, 0.44; 95% CI, 0.30 to 0.58). The hospital-level LHS was most negatively associated with the South Census region (coefficient, −0.13; 95% CI, −0.26 to −0.004) (Table 3).

**Table 3.** Univariate Regressions to Test the Association Between Local Hospital Segregation Index and Hospital Characteristics Using Center for Medicare & Medicaid Services Data From 2019

Hospital characteristic	Coefficient (95% CI)
Model 1: % Black residents in area, per 10 percentage points	0.15 (0.11-0.19)
Model 2: market share	
≤2	0 [Reference]
3 to 6	−0.09 (−0.22 to 0.05)
≥7	0.18 (0.05-0.31)
Model 3: hospital ownership	
Nonprofit	0 [Reference]
Physician or other	0.16 (0.004 to 0.31)
Private (for profit)	0.04 (−0.08 to 0.15)
Government	0.24 (0.10 to 0.38)
Model 4: teaching hospital^a^	
Nonteaching hospital	0 [Reference]
Teaching hospital	0.36 (0.28 to 0.45)
Model 5: disproportionate share hospital^b^	
No	0 [Reference]
Yes	0.44 (0.30 to 0.58)
Model 6: census region	
Northeast	0 [Reference]
Midwest	−0.09 (−0.24 to 0.06)
South	−0.13 (−0.26 to −0.004)
West	−0.07 (−0.22 to 0.08)

^a^ Seven hospitals were missing data on this variable and were excluded from this analysis.

^b^ Disproportionate share hospitals are hospitals that receive government funding to provide care to uninsured patients.

## Discussion

This cross-sectional study assessed the differential sorting of Black patients into hospitals within a geographic region based on representativeness of hospitalizations by race within that region. There is a long-standing literature on the association between residential segregation and the quality of health care.^1,2,18,19^ We found that differential sorting, as measured by the LHS index, was common, particularly in areas with large concentrations of Black people. We also found that the LHS index at the hospital level was significantly associated with the hospital being a government (likely safety net) hospital, teaching hospital status, and receiving DSH payments.

Differential access to health care services and variations in health care quality have historically and contemporarily contributed to the health disparities demonstrated in Black patients in the United States.^2,4,6^ Prior to the Civil Rights Act of 1964, Black patients were not allowed to be treated in segregated hospitals.^20^ The Civil Rights Act of 1964 made segregation illegal and theoretically removed a large barrier to accessing health care for Black patients, but it was not until the 1966 implementation of Medicare that hospitals truly began to integrate.^20^ Despite there no longer being laws that can legally prevent Black patients from being seen in specific hospitals, our study provides evidence that there remains a sorting of patients by which Black patients go to one hospital and White patients another, even if patients need to travel farther as a consequence.^21^ This pattern suggests an alternate explanation that may be related to structures and protocols at the hospital level that need to be further explored.

There is considerable evidence that residential segregation drives variation in hospital racial composition, with several recent studies showing poor hospital quality in residentially segregated areas.^8,9^ This, in part, reflects the lower quality of hospitals located in largely Black communities. While there is much less evidence on how quality of care is affected, a 2022 study found that Black patients were more likely to receive care at lower-performing hospitals than White patients, even when they lived in the same zip code or HRR.^22^ Using the HRR as a measure of the market, a study by Lin et al^8^ also found considerable patient sorting by race at the regional level.

In recent years, there have been 2 main approaches to measure inclusivity conditional on the degree of racial segregation.^11^ One approach uses Dartmouth hospital service areas (HSAs, of which there are >3000 in the US) as hospital markets rather than driving time, but these service areas vary dramatically in size (for example, Los Angeles, California, is a single HSA).^23^ The other approach defines a hospital’s market based on its current patient composition (which may, in turn, reflect racial sorting), rather than our predetermined 30-minute drive time.^10^ In any case, it is apparent that further investigation is needed to understand what institutional and structural mechanisms may be contributing to these differences, and how quality of care may be affected by patient sorting. We acknowledge there are a variety of documented reasons why patients may choose different—and, in some cases, more distant—hospitals^17^ that are not captured in this analysis.

### Limitations

There are limitations to this study. First, a 30-minute driving distance of hospital markets may be too large in cities and too small in rural areas. In addition, drive time does not account for use of public transportation. To address this concern, we considered 15-minute and 1-hour driving distances; while estimates of positive values of the LHS were robust to how the market is defined, local neighborhood factors may matter more for negative values of the LHS. Second, applying a single definition of market area to different geographic areas may be restrictive, but this approach is commonly used to avoid market measures that are themselves affected by the outcome variable^24^ (eAppendix in Supplement 1). Third, the sample is limited to Medicare FFS patients, rather than all regional residents who rely on local hospitals (Medicaid- and commercially insured pediatric and maternity care, other commercially insured populations, Medicare Advantage, and the uninsured). For example, Medicare Advantage now accounts for more than half of Black Medicare enrollees.^25^ However, the Medicare Advantage encounter data are not uniformly reliable^26^ and often institute narrow networks with higher copays for out-of-network hospitals, thus potentially biasing results. Additionally, the LHS index does not adjust for factors such as whether the admission is urgent or elective or the medical or surgical condition for which the patient is admitted.

## Conclusion

In this cross-sectional study using a national sample of acute care hospitals in 2019, we used a novel measure of LHS to capture the sorting of patients who lived in the same markets to different hospitals. The LHS index could be leveraged by policy makers and clinical leaders to inform payment reform and hospital quality scores, thereby increasing equitable access to high-quality hospitals.

## Notes

Supplement 1. **eAppendix.** Supplementary Methods and Sensitivity Analysis

Supplement 2.
Data Sharing Statement
