# Supplementary material for: Measuring Local-Area Racial Segregation for Medicare Hospital Admissions
Source: JAMA Netw Open. 2024 Apr 19;7(4):e247473. doi: 10.1001/jamanetworkopen.2024.7473 (PMC11031679; doi:10.1001/jamanetworkopen.2024.7473)
Supplement: Supplement 1. — eAppendix. Supplementary Methods and Sensitivity Analysis [file jamanetwopen-e247473-s001.pdf]

Supplemental Online Content

Akré EL, Chyn D, Carlos HA, Barnato AE, Skinner J. Measuring local-area racial segregation for Medicare hospital admissions. *JAMA Netw Open*. 2024;7(4):e247473. doi:10.1001/jamanetworkopen.2024.7473

eAppendix. Supplementary Methods and Sensitivity Analysis

This supplemental material has been provided by the authors to give readers additional information about their work.

© 2024 Akré EL et al. *JAMA Network Open.*

Notes on the LHS Index and the Regional LHS Index

In this section we consider in more detail the Local Hospital Segregation (LHS) index and the generalization of the LHS index across Hospital Referral Regions (HRRs) (and the way in which our measure differs from both Lin et al. (2023) and the Lown Institute Inclusivity measure.) We then consider the complications when there are several hospital markets that overlap in a given ZIP code. Finally, we describe the regional measure defined over the HRR.

A.1.a. As noted in the methods section, we seek to develop a measure of the LHS that uses as its potential market a region that is "exogenous"--or, not dependent on patient preferences or hospital strategies--to attract patients from one ZIP code but not another. For this reason, we follow the literature (e.g., Dauda, 2018) by using fixed driving times as the market definition. Although defining the market using ZIP codes of people who are admitted to that hospital (as in the Lown Institute (2022) approach) may provide an accurate measure of where the hospital is drawing its patients, the market becomes a function of both patient and hospital (marketing) choices resulting in the observed admission patterns at the hospital. The Lin et al. (2023) measure is also a valuable way to measure misallocation across the HRR, but it is defined only at the HRR level and not at the hospital level. While the definition of the HRR is designed to capture "tertiary" care (such as cardiac surgery or neurosurgery), travel costs for traveling across HRRs could be quite large especially for more common hospital admissions. (We acknowledge that hospitals often draw patients from far beyond 30-minute or 60-minute driving distances, which is why we focus on the subset of a hospital's patients within the driving limit.) Finally, the *US News and World Report* measure (Binger et al., 2022), which is restricted to publicly reported data, uses hospital service areas, many of which are small (and some of which are very large, such as Los Angeles) and lack more than one hospital; furthermore, they must impute the denominator for their calculations from county-level data. Also see Golden and Powe (2023) for an excellent discussion of such measures.

A.1.b. While the intuition of an LHS index is straightforward, complications arise when two or more hospitals exhibit overlapping markets. Figure A.1 shows a hypothetical city with two hospitals, A and C, one of which is twice as large (in terms of hospitalizations) as the other. Each Medicare hospitalization is denoted as either B (Black) or N (people of other racial or ethnic identities than Black, denoted here as not black). The

© 2024 Akré EL et al. *JAMA Network Open.*

dashed lines denote our hypothetical 30-minute driving radius around each hospital; note that patient hospitalizations to the left of Hospital A are of patients only in Hospital A's market, while hospitalizations to the right of Hospital C are of patients only in Hospital C's market. People located between the hospitals are among patients residing in both hospitals' markets.

Figure A.1: Schematic of Market Shares when Hospitals Share Neighborhoods.

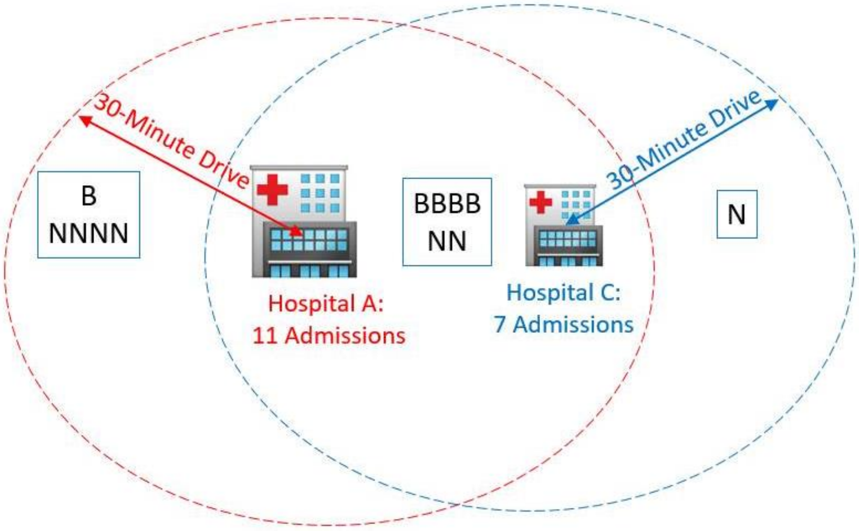

One might construct the community-level distribution of Hospital C's market by adding up all hospitalizations inside the blue circle in Figure A.1; this corresponds to a total of seven, of which four are Black and three are Not Black (or 57% Black); the equivalent ratio for Hospital A is 45% (5/11).

However, it is likely that both hospitals will fall short of their "market" racial distribution since they share six hospitalizations (four Black and two Not Black); thus, it's impossible for *both* hospitals to treat 57% and 45% Black patients, respectively. In practice, however, we do not find evidence of this bias, since the mean value of the LHS across markets is generally zero (rather than being negative, as would be suggested by this example).

A.1.c. We wish to create a measure that addresses the question: what fraction of total hospitalizations in the regional market (the HRR in our case) must have been admitted to a different hospital to assure "evenness" or the hospital's fraction of Black hospitalizations equal to the hospital market's fraction of Black residents?

This of course is very much in the tradition of the dissimilarity index (see, for example, Lin et al., 2023), but there is a difference between the two. The dissimilarity index measures how many Black residents (for example) would move to attain residential evenness; in this case, we are holding the total number of hospitalizations at each hospital constant, so we would need to hypothetically "swap" both a Black patient and an offsetting non-Black patient (or if the denominator were only Black and White, a White patient) to keep hospitalizations constant at each hospital.

Briefly, our regional measure is a weighted average of the absolute value of the LHS, where the weight is the fraction of the total HRR hospitalizations at hospital i. It's easiest to see this in an example. Suppose there are three hospitals (and let's ignore inflows and outflows). Hospitals A, B and C have 50, 100 and 200 hospitalizations, respectively, for a total of 350 hospitalizations. Suppose 10% (35) of these are among Black patients. Twenty patients are admitted to Hospital A, 10 to Hospital B and five to Hospital C.

The LHS for Hospital A is 0.30 ((20-5)/50); for Hospital B it's zero ((10-10)/100); and for C it's -0.075 ((5-20)/200). Note that, because of the uneven sizes, they do not add to zero. But the number of hospitalizations needing to have occurred at a different hospital to attain "evenness" is 15 among Black patients (admitted to Hospital A rather than Hospital C) and 15 among patients who do not identify as Black (admitted to Hospital C rather than Hospital A). So conceptually, the regional "dissimilarity" index should end up being 30/350 = 0.0857. This is equal to the weighted average (where the hospital weights for A, B and C are .143, .286 and .571) of the absolute values of the LHS (0.30, 0, -0.075).

A.2 Sample Composition

In Figure A.2, we outline how our dataset was constructed, keeping track of both the number of hospitals and the number of patients thus excluded. We chose to focus on hospitals with at least 200 admissions to ensure greater statistical precision. Regarding the last step in Figure A.2, the CMS suppression policy for reporting data on beneficiaries stipulates that counts of patients between 1 and 10 cannot be reported directly; this policy also applies in cases when a count of 1-10 patients could be derived when used in combination with other reported information. We make this explicit restriction in our data build in the form of excluding hospitals that treated fewer than 11 Black patients or fewer than 11 patients not racialized as Black in 2019. We further

© 2024 Akré EL et al. *JAMA Network Open.*

applied this restriction after redefining our sample hospitals to consider all 65+ Medicare FFS patients (vs just those racialized as Black or White, as was done in earlier work). For a few hospitals, these preliminary (unpublished) counts could be used in conjunction with the expanded counts to derive a count of patients identified as neither Black nor White between 1 and 10. Therefore, we additionally excluded these hospitals from the final (published) analysis presented in this paper.

© 2024 Akré EL et al. *JAMA Network Open.*

Figure A.2: Sample Composition

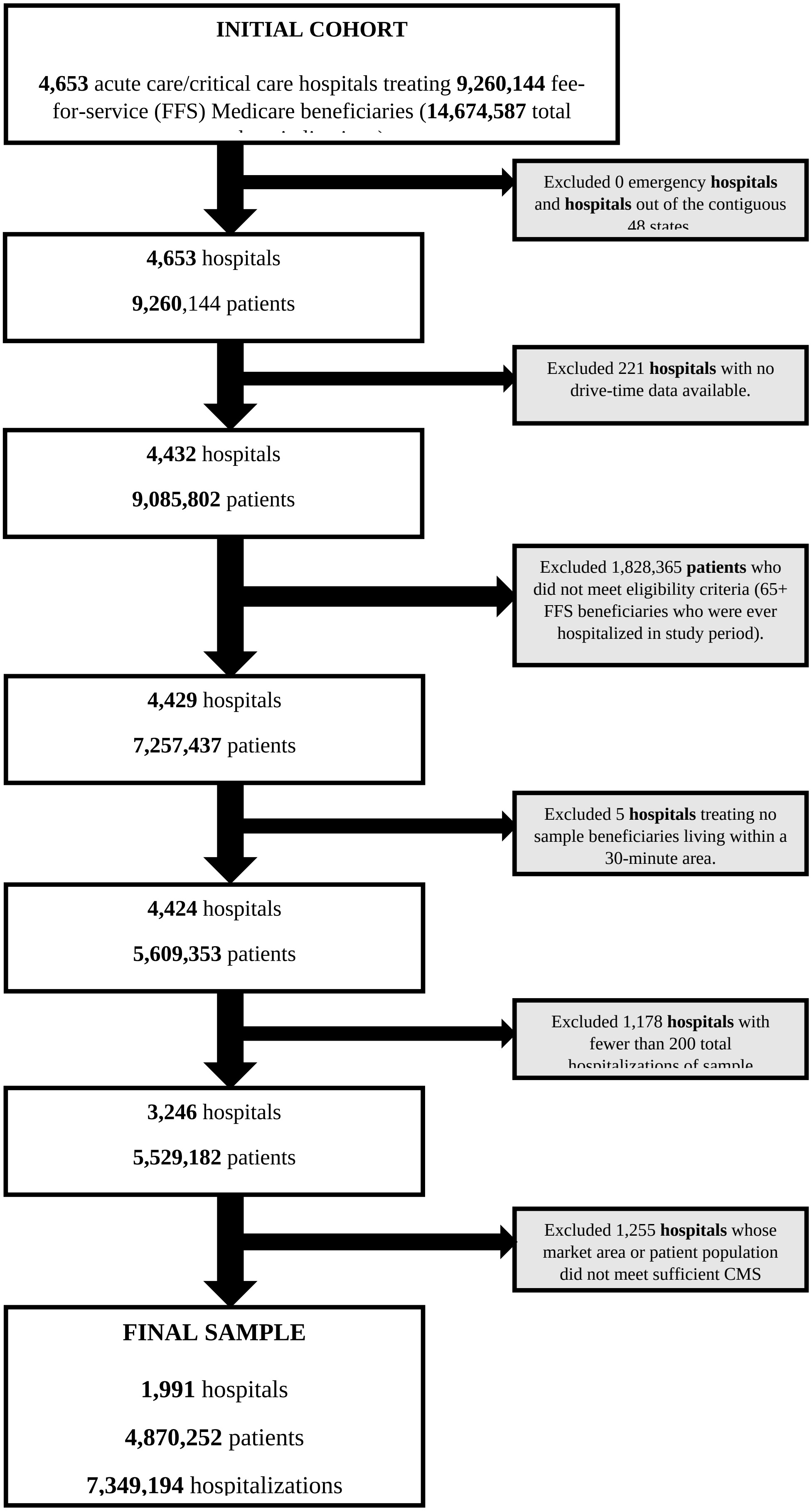

© 2024 Akré EL et al. *JAMA Network Open.*

A.3 Sensitivity Analysis for Regional LHS Scores by Drive Times

Figure A.3.1: Scatter Plot of HRRs by the Percent Black Hospitalization in the HRR and the Regional Local Hospital Segregation Index for the HRR: Market Defined by 15-Minute Driving Radius

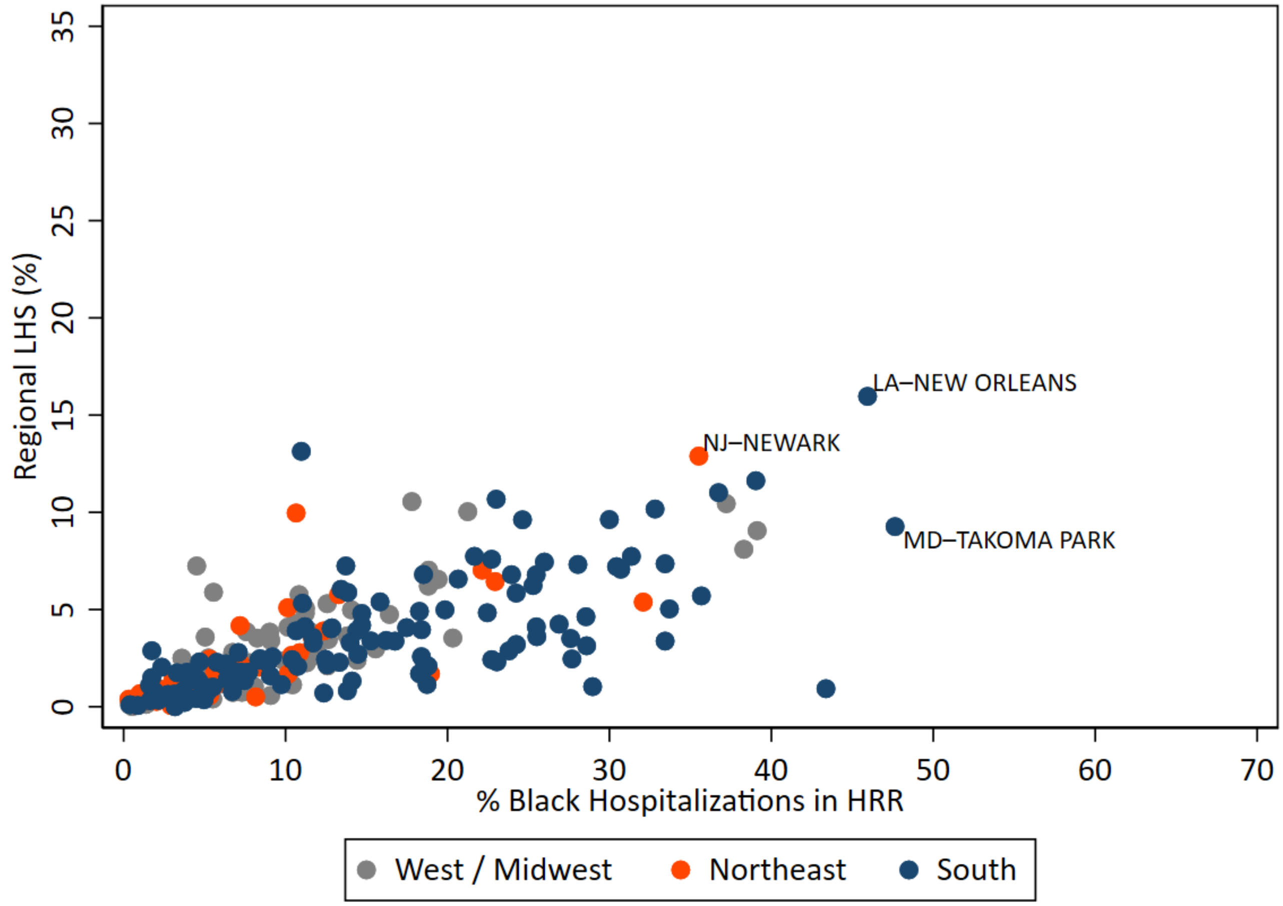

While the measures are correlated, there is still considerable variation regarding the regional LHS holding constant the percent of Black hospitalizations in the HRR. In general, the regional LHS index for the 15-minute radius is of smaller magnitude. Source: 2019 CMS Medicare data.

© 2024 Akré EL et al. *JAMA Network Open.*

Figure A.3.2: Scatter Plot of HRRs by the Percent Black Hospitalization in the HRR and the Regional Local Hospital Segregation Index for the HRR: Market Defined by 60-Minute Driving Radius

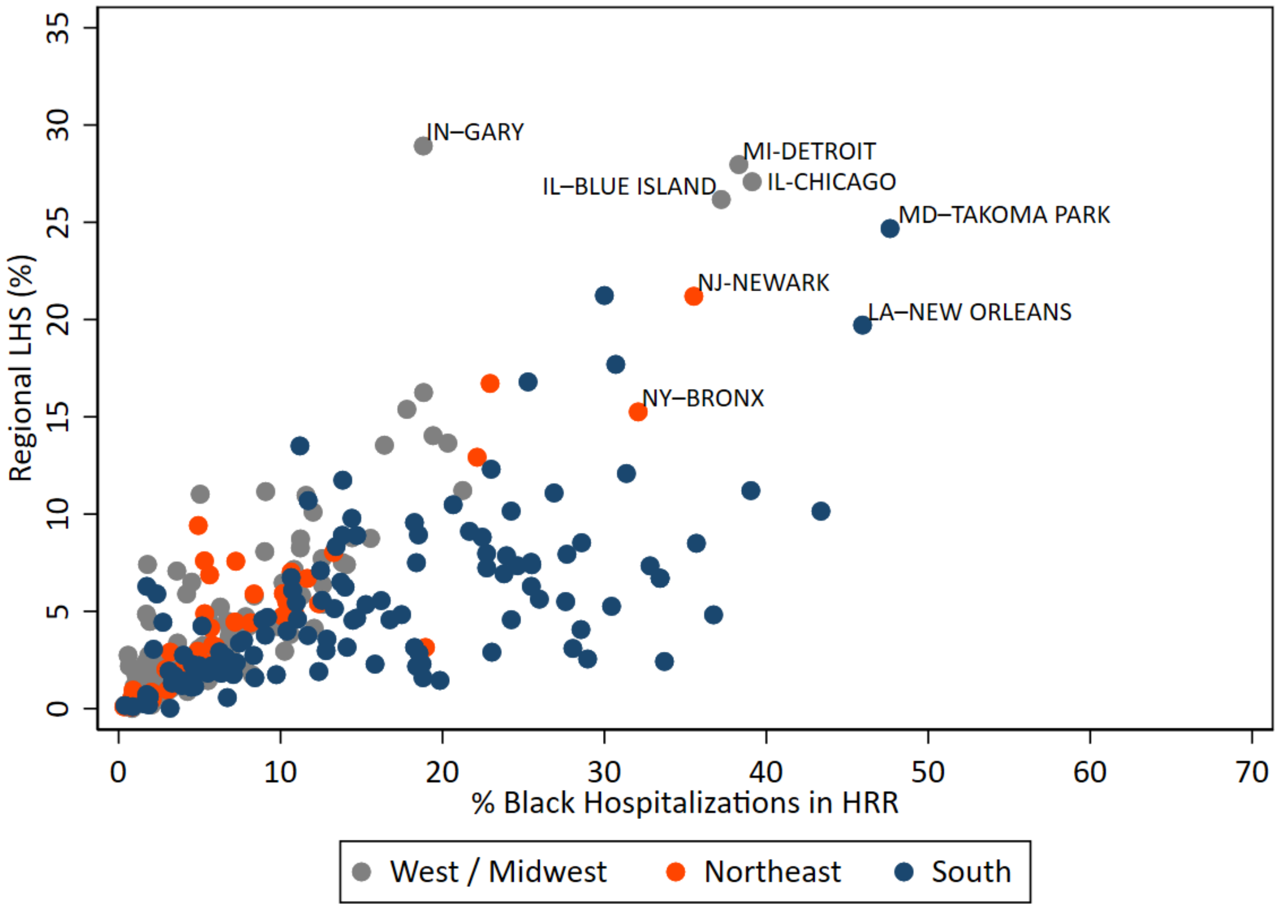

While the measures are correlated, there is still considerable variation with regard to the regional LHS holding constant the percent of Black hospitalizations in the HRR. Source: 2019 CMS Medicare data.

© 2024 Akré EL et al. *JAMA Network Open.*

References

Binger T, Chen H, Adams Z, et al. 2022. Methodology: US News &World Report 2022-2023 best hospitals health equity measures. US News &World Report. July 26. Accessed December 10, 2023. https://health.usnews.com/media/best-hospitals/Best-Hospitals-Health-Equity-2022-23

Dauda, S., 2018. "Hospital and Health Insurance Markets Concentration and Inpatient Hospital Transaction Prices in the US Health Care Market," Health Services Research, 53(2), pp.1203-1226.

Golden, S.H., and N. R. Powe, 2023. "Hospital Equity Rating Metrics—Promise, Pitfalls, and Peril," JAMA Health Forum 2023;4(10): e233188. doi:10.1001/jamahealthforum.2023.3188

Lin, S.C., Hammond, G, Esposito, M., Majewski, C., Foraker, R.E., and K. E. Joynt Maddox, 2023. "Segregated Patterns of Hospital Care Delivery and Health Outcomes." JAMA Health Forum,

Lown Institute, 2022. https://lownhospitalsindex.org/rankings/our-methodology/ Accessed December 11,, 2023.

© 2024 Akré EL et al. *JAMA Network Open.*
