# Supplementary material for: Measuring Local-Area Racial Segregation for Medicare Hospital Admissions
Source: JAMA Netw Open. 2024 Apr 19;7(4):e247473. doi: 10.1001/jamanetworkopen.2024.7473 (PMC11031679; doi:10.1001/jamanetworkopen.2024.7473)
Supplement: Supplement 2. — Data Sharing Statement [file jamanetwopen-e247473-s002.pdf]

Data Sharing Statement

Akré. Measuring Local-Area Racial Segregation for Medicare Hospital Admissions. *JAMA Netw Open*. Published April 19, 2024. doi:10.1001/jamanetworkopen.2024.7473

Data
Data available: No

Additional Information
Explanation for why data not available: Data requires a CMS DUA
